# Responder signatures and predictors of upper- and lower-limb power responsiveness to maximal strength versus plyometric dry-land training in swimmers

**DOI:** 10.3389/fphys.2026.1799253

**Published:** 2026-03-04

**Authors:** Liuxi Yang, Qi Xu, Yu Chen, Jiushu Zhou

**Affiliations:** 1 College of Aviation, Civil Aviation Flight University of China, Guanghan, China; 2 Gdansk University of Physical Education and Sport, Gdańsk, Poland; 3 School of Physical Education, Chengdu Sport University, Chengdu, China

**Keywords:** dry-land training, hierarchicalclustering, inter-individual variability, neuromuscular power, responder classification, strengthand conditioning, swimming performance

## Abstract

**Objectives:**

To quantify upper- and lower-limb power responsiveness to maximal strength (MSTG) versus plyometric training (PTG) versus control (CG), and to identify stable responder signatures from the 2D change vector (ΔUpper, ΔLower).

**Methods:**

Twenty-seven university swimmers were randomized to MSTG, PTG, or CG (n = 9 each) for 6 weeks with testing at Pre, Mid, and Post. Upper- and lower-limb power constructs were derived via baseline-fitted PCA from bench press power plus medicine-ball throw and from CMJ, SJ, DJ, plus SLJ, respectively; responsiveness was Post–Pre. Group contrasts used permutation tests with Holm adjustment and bootstrap confidence intervals. Responder signatures were identified by Ward clustering with cluster-number selection and bootstrap stability.

**Results:**

ΔUpper was 0.962 ± 0.129 (MSTG), 0.762 ± 0.218 (PTG), and 0.332 ± 0.058 (CG); MSTG–PTG mean difference was 0.200 (95% CI [0.047, 0.356], p = 0.030, g = 1.065), and both exceeded CG (p < 0.001). ΔLower was 0.822 ± 0.125 (MSTG), 0.758 ± 0.150 (PTG), and 0.388 ± 0.059 (CG); MSTG–PTG was 0.065 (p = 0.331), while both exceeded CG (p < 0.001). Clustering selected k = 2 (silhouette 0.608) with high stability (ARI 0.840 [0.591, 1.000]) and strong group association (χ^2^ = 18.900, p < 0.001).

**Conclusion:**

In this sample, MSTG elicited larger upper-limb responsiveness than PTG, while both approaches improved upper- and lower-limb constructs versus CG, and responder signatures were stable and strongly aligned with training modality. In exploratory models within our sample, short-duration intervention, training allocation (stimulus) dominated modeled responsiveness and baseline sprint performance showed an inverse association with high-responder membership, patterns consistent with short-block trainability/ceiling effects, therefore these predictive findings should be interpreted as context-specific and not generalized beyond similar swimmer levels and intervention doses.

## 1 Introduction

Competitive sprint swimming performance is strongly influenced by the ability to generate high propulsive impulses and to rapidly accelerate the body during starts and turns, which has positioned dry-land strength and power training as a central complement to in-water practice in many programs (Crowley et al., 2017). Resistance training is widely used to target neuromuscular qualities that are difficult to overload sufficiently in water, and systematic reviews indicate that appropriately prescribed resistance training can improve swimming performance and related determinants (Crowley et al., 2017; Fone and van den Tillaar, 2022). Plyometric approaches are also commonly implemented to enhance stretch–shortening cycle function and explosive lower-limb output, with evidence supporting improvements in swim start and turn-related performance following plyometric interventions (Bishop et al., 2009).

The state of the art shows that dry-land resistance training meaningfully improves strength-related qualities and can translate to faster swim performance across sprint and middle distances, although the magnitude of transfer varies across studies and training modalities (Crowley et al., 2017). Recent syntheses further suggest that the methodological characteristics of resistance training programs, including exercise selection and emphasis on strength versus power, can differentially affect outcomes in competitive swimmers (Jin et al., 2024). Similarly, controlled and quasi-controlled work indicates that plyometric training can improve jump-based proxies of power and sport-specific swimming performance, including start-related metrics and sprint times (Bishop et al., 2009; Sammoud et al., 2019).

Despite this progress, much of the literature remains focused on group-average effects and isolated endpoints, which can obscure meaningful inter-individual variability in adaptation and limit the translation to individualized programming (Ross et al., 2019; Bonafiglia et al., 2021). Contemporary methodological reviews highlight that “responder” classification is highly sensitive to statistical choices, outcome selection, and measurement error, and they recommend rigorous approaches when quantifying inter-individual differences in trainability (Bonafiglia et al., 2021). In swimmers specifically, reviews of dry-land interventions for starts and turns note substantial heterogeneity in training content and outcome selection, which complicates direct comparison across modalities and may mask distinct profiles of adaptation (Hermosilla et al., 2021).

Addressing these gaps requires analytical frameworks that integrate multiple neuromuscular indicators into interpretable constructs, quantify adaptations as multivariate change patterns, and formally evaluate the stability of responder classifications (Ross et al., 2019; Bonafiglia et al., 2021). Moreover, because variability in training response is now recognized as a reproducible biological and methodological phenomenon across exercise domains, identifying predictors of responsiveness represents a practical step toward precision-oriented training prescription (Ross et al., 2019), however, predictive signals are highly contingent on athlete level, intervention dose, and outcome definitions, and require external validation before any broad monitoring or talent-development application is implied. In competitive swimming, this integrated approach is particularly relevant because dry-land improvements may transfer differently across upper-versus lower-limb contributions, and because start and turn determinants can be influenced by distinct strength–power qualities (Bishop et al., 2009; Hermosilla et al., 2021).

Accordingly, the objectives of the present study were to quantify training-induced changes in upper- and lower-limb power constructs derived from multiple performance indicators and to compare responsiveness between training approaches using post–pre change scores. The study further aimed to identify stable responder signatures by clustering individuals in the two-dimensional change space defined by ΔUpper (Post–Pre) and ΔLower (Post–Pre), including explicit cluster-number selection and stability evaluation. Finally, the study aimed to exploratorily model predictors of responsiveness and responder-signature membership, examining the contribution of training modality and baseline characteristics to inter-individual variation in adaptation within this sample and intervention dose.

## 2 Methods

### 2.1 Experimental approach

This study was a three-arm, parallel-group randomized controlled trial conducted in a university swimming program. Participants completed their usual in-water training supervised by the team coaches and, in addition, undertook a 6-week dry-land intervention delivered twice per week. The two experimental interventions were maximal strength training (MSTG) and plyometric training (PTG), and the active control condition was muscular endurance training (CG) representing the dry-land routine commonly used in the team. Assessments were performed at three standardized time points: 1 week before the start of the intervention (Pre), at the end of week three (Mid), and in the week immediately after completion of week six (Post). Testing days, test order, and environmental conditions were kept consistent across assessment waves, and participants refrained from strenuous exercise for 48 h before each evaluation.

Randomization used a 1:1:1 allocation to MSTG, PTG, or CG. To reduce potential confounding from sex-specific training backgrounds, assignment was performed within sex strata. The random sequence was implemented through an envelope-based procedure in which opaque, sealed envelopes were distributed immediately prior to baseline assessment, ensuring allocation concealment until assignment. A researcher not involved in outcome testing administered the randomization process. Outcome assessors were independent of the training delivery team and were blinded to group allocation and intervention details. Swimmers and coaches could not be blinded because of the obvious differences in training modality.

### 2.2 Participants

Swimmers were recruited from a local university team through direct contact with coaches and team staff. Eligibility required attendance at all three assessment moments, at least 5 years of swimming experience and at least 2 years of resistance-training experience, completion of at least 90% of the prescribed dry-land sessions, no injury or illness during the intervention or the preceding month, and no concurrent participation in other strength and conditioning programs. Exclusion criteria were non-attendance at any assessment or use of substances likely to affect training adaptations. Eligibility was confirmed independently by two researchers with doctoral training in sport science and more than 5 years of applied strength and conditioning experience.

*A priori* sample size estimation was performed in G*Power (version 3.1.9, Universität Düsseldorf, Germany) for the within-between interaction in repeated-measures models (power 0.95, α 0.05), using an effect size informed by prior work comparing plyometric training effects on 25 m front crawl performance. Thirty swimmers were initially enrolled; three were withdrawn due to unrelated injuries sustained outside the intervention, leaving twenty-seven swimmers (10 women, 17 men) for analysis.

Across the 27 swimmers included in the analyses, 10 were women and 17 were men, with comparable sex distribution across groups, including 3 women and 6 men in the maximal strength training group, 4 women and 5 men in the plyometric training group, and 3 women and 6 men in the muscular endurance training group. Overall, participants were 20.2 ± 1.1 years old and reported 9.1 ± 1.5 years of swimming experience; corresponding values were 20.0 ± 1.0 years and 9.1 ± 1.7 years in MSTG, 20.4 ± 1.3 years and 9.2 ± 1.5 years in PTG, and 20.2 ± 1.0 years and 8.9 ± 1.5 years in CG. Mean stature was 177.9 ± 9.0 cm in the full sample, with group values of 180.3 ± 6.6 cm for MSTG, 176.2 ± 9.8 cm for PTG, and 177.1 ± 10.7 cm for CG, while body mass averaged 69.9 ± 13.7 kg overall, with 75.0 ± 16.0 kg in MSTG, 66.2 ± 11.1 kg in PTG, and 68.3 ± 13.7 kg in CG. Body mass index was 22.0 ± 3.3 kg/m^2^ overall, including 23.0 ± 4.6 kg/m^2^ in MSTG, 21.2 ± 2.2 kg/m^2^ in PTG, and 21.7 ± 2.7 kg/m^2^ in CG. Best 50 m freestyle performance was 27.9 ± 1.8 s for the full sample, with group means of 27.2 ± 1.6 s in MSTG, 28.0 ± 2.0 s in PTG, and 28.5 ± 1.9 s in CG.

Participants were categorized as Tier 2 (trained/developmental) according to the Participant Classification Framework (McKay et al., 2022), reflecting regular regional-level competition exposure, three in-water sessions per week, and approximately 3 h and 30 min of weekly swimming training time, accordingly, this sample should be interpreted as developmental/trained rather than highly trained/elite (Tier 3–5), and the magnitude of short-term adaptations observed here may not generalize to higher-tier swimmers. The in-water program was planned and delivered exclusively by team coaches without input from the research group.

The protocol was approved by the Ethics Committee of Chengdu Sport University (approval code 120-20240913). All procedures complied with the Declaration of Helsinki. Prior to enrolment, swimmers received written and verbal information about the study purpose, procedures, and risks, and provided written informed consent. Participation was voluntary and could be discontinued at any time without consequence.

### 2.3 Interventions

All participants continued their habitual swimming training under the supervision of their coaches. The typical structure included warm-up sets, technical drills, and conditioning work such as high-intensity interval training or sprint sets followed by cooldown. Dry-land interventions were performed on two non-consecutive days each week (Mondays and Thursdays) for 6 weeks, totaling 12 sessions, which represents a relatively low-dose, short-duration exposure likely to accentuate early/novelty-driven adaptations in trained/developmental swimmers. Sessions lasted approximately 50–60 min, including 10–15 min of preparatory activities. Dry-land training and swimming sessions were scheduled separately to support recovery.

Across groups, the progression strategy increased the overall training challenge during weeks 1–5 and reduced loading in week 6 to facilitate recovery and consolidation of adaptations. Each session began with approximately 5 minutes of light running followed by dynamic mobility and stretching targeting upper and lower limbs.

#### 2.3.1 Maximal strength training (MSTG)

The MSTG program emphasized high-load, multi-joint barbell exercises: bench press, back squat, and deadlift. Loads were prescribed relative to one-repetition maximum (1RM) and adjusted to maintain technical quality while achieving near-failure within the target repetition ranges. The weekly intensity–repetition progression followed an 80%–85% 1RM range for 6–8 repetitions in week 1, 85%–90% 1RM for 4–6 repetitions in weeks 2–3, 90%–95% 1RM for 2–4 repetitions in weeks 4–5, and a taper week (week 6) returning to 85%–90% 1RM for 4–6 repetitions. Rest intervals were 3–4 min between sets. When an athlete repeatedly reached the top of the prescribed repetition range with good form, the subsequent load was increased by 2.5 kg.

Technique was standardized across participants. During bench press, athletes maintained a five-point contact position with the head, shoulders, pelvis, and both feet in contact with the bench, avoided any bar bounce, and controlled the eccentric phase with the elbows oriented at approximately 45°. During back squat, a high-bar position was used and depth was standardized to a knee flexion angle below 90°, with a controlled eccentric and an intent to accelerate rapidly during the concentric phase. Deadlifts were executed with an emphasis on explosive intent, reflecting the rapid force production demands of the swimming start. Sessions were supervised by a qualified strength and conditioning coach.

#### 2.3.2 Plyometric training (PTG)

The PTG program targeted rapid stretch–shortening cycle actions and horizontal-to-vertical force transfer relevant to starts and sprint swimming. Upper-limb plyometrics consisted of explosive push-ups and two-hand forward medicine-ball throws. Lower-limb drills included mixed-direction jumps (e.g., alternating-leg jumps with arm swing, explosive mat jumps, hurdle-style continuous jumps, and continuous jumps) and vertical-direction jumps (e.g., alternating-leg kicks, split-leg squat jumps, and longitudinal jumps). Each session included five exercises comprising two upper-limb drills, two mixed-direction lower-limb drills, and one vertical-direction drill. Split squats were incorporated to reinforce start-specific neuromuscular recruitment patterns.

All repetitions were performed with maximal intent and maximal movement speed. All repetitions were performed with maximal intent and maximal movement speed. To promote true stretch–shortening cycle (SSC) execution, lower-limb drills were consistently cued as “rebound immediately” and “minimize ground contact time,” and supervisors provided real-time feedback on landing stiffness, alignment, and rapid coupling between eccentric landing and concentric take-off. However, we did not instrument training sessions with objective kinematic or contact-time monitoring (e.g., force plates, optical systems, or wearable sensors). Between-set and between-exercise recovery was standardized to 1–2 min. Contact volume progressed during weeks 1–5, and was modestly reduced during week 6. Warm-up contacts were not counted toward the prescribed totals.

#### 2.3.3 Control group (CG)

The CG condition represented an active control emphasizing moderate-intensity, higher-repetition resistance exercises. The program comprised five exercises performed for 4 sets of 15 repetitions each: push-ups, standing sleeper support, dumbbell bird raises with 3 kg, bodyweight squats, and an upper-body pulling movement (men performed pull-ups; women performed an oblique pulling variant). Set recovery was 2–3 min, and movement tempo was controlled to prioritize consistent execution.

For the pulling exercise, participants used a shoulder-width grip with the arms positioned at approximately a 90-degree angle relative to the torso. Repetitions were counted when the chin reached or cleared the bar, followed by a controlled return to the starting position.

To promote fidelity and safety, each group trained under the supervision of a dedicated researcher or assistant with at least 3 years of experience in strength and conditioning coaching. Supervisors provided real-time technical feedback and standardized verbal encouragement to ensure high effort and adherence to the prescribed execution standards across all sessions.

### 2.4 Outcome measures and testing procedures

All assessments were performed in the morning. Testing began in a climate-controlled room maintained at 22 °C and 55% relative humidity, where demographic information, anthropometrics, and strength levels were recorded (strength tests were used for training-load adjustment). Athletes then completed standardized in-water tests in a 50 m pool. The in-water warm-up consisted of dynamic movements for upper and lower limbs, followed by a 600 m freestyle swim and three 50 m freestyle accelerations at low-to-moderate intensity. After 3 minutes of passive recovery, swimming tests commenced.

Swimming performance tests were administered in a fixed sequence. The start phase was assessed by flight distance and platform-to-15 m time using a standardized kick-start technique with back-foot tilt. Flight distance was quantified as the horizontal distance from the start wall to the fingertip water-entry point. Video was captured using an iPhone 14 (1080p at 30 fps) positioned 3.5 m from the pool edge, parallel to the lane, at a height of 130 cm with the lower frame aligned to the waterline. Kinovea software (version 0.9.5-x64) was used for two-dimensional analysis with calibration using fixed-length poles placed relative to the start wall. Platform-to-15 m time was defined from the start signal to the moment the swimmer’s head reached the 15 m mark.

Swimming-specific sprint performance was assessed with four timed trials: 25 m freestyle kick without arm strokes, 25 m freestyle arm stroke without kicking, 25 m freestyle front crawl, and 50 m freestyle. For 25 m trials, time was measured from the start signal until the swimmer’s head reached the 25 m mark; for 50 m, time was measured from the signal until fingertip contact with the wall at 50 m. Pool markings at 15 m, 25 m, and 50 m standardized distance identification. Each test was performed once, and times were recorded with an electronic stopwatch (LI-NING 019-1, China) by the same experienced evaluator. All trials were video-recorded (iPhone 14, 1080p/30 fps) to verify time points, synchronized to the start signal via a light trigger, and reviewed using SwimWatch Race Analyzer (NatriSoft, Netherlands). A pilot reliability check comparing stopwatch with video-based timing yielded an intraclass correlation coefficient of 0.93. Ten minutes of rest separated the sprint tests to support full recovery.

#### 2.4.1 Land-based power assessments

Upper-limb power was assessed with average concentric power during bench press and with an overhead medicine-ball throw. For bench press power, a standardized barbell load was used (30 kg for men and 20 kg for women). Participants assumed a stable position, gripped the bar slightly wider than shoulder width, and performed the lift on the tester’s command. The bar was lowered under control to lightly touch the chest before being pressed upward as fast as possible without bouncing; elbow orientation was standardized to approximately 45° during the eccentric phase. Three trials were performed with at least 2 minutes of rest, and average maximal power (W) was recorded with a GYKO power measurement system (Microgate, Italy).

Medicine-ball throw performance was assessed as maximal throwing distance using a standing staggered stance behind a line. Participants raised the ball overhead with full upper-limb extension and released at approximately a 45-degree trajectory with maximal speed. Throw distance was determined with Kinovea (version 0.9.5-x64) using a 100 cm calibration reference visible during recording. Three trials were completed with at least 2 minutes of recovery, and the best distance was retained.

Lower-limb power was assessed using countermovement jump (CMJ), squat jump (SJ), drop jump (DJ), and standing long jump (SLJ). Jump height for CMJ, SJ, and DJ was captured with OptoJump (Microgate, Italy). For CMJ, participants kept hands on the waist, descended to approximately parallel thigh position, and jumped maximally. For SJ, the same position was held for 2 seconds to minimize countermovement before the maximal jump. For DJ, participants stepped from a 40 cm box without actively jumping down, landed with both feet, and immediately rebounded upward. SLJ distance was obtained via Kinovea with a 100 cm calibration reference; participants jumped horizontally from behind a start line, landed with both feet, and avoided falling forward or backward. Each test involved three trials separated by at least 2 minutes, with the best trial used for analysis.

#### 2.4.2 Data preparation

To align outcomes with the construct-based and responder-signature objectives of the present report, upper- and lower-limb power composites were defined *a priori* from the land-based indicators. Upper-limb power was operationalized using bench press average power and medicine-ball throw distance, and lower-limb power was operationalized using CMJ, SJ, DJ, and SLJ. Indicators were standardized using baseline means and standard deviations, and principal component analysis was fitted at baseline within each domain to derive a single composite score; fixed baseline loadings were then applied to compute composite values at Pre, Mid, and Post. Responsiveness was quantified as Post–Pre change scores for upper-limb and lower-limb composites (ΔUpper and ΔLower). Responder signatures were derived by clustering the two-dimensional change vector (ΔUpper, ΔLower) with cluster-number selection and stability checks.

### 2.5 Statistical procedures

All analyses were conducted in Python using pandas and numpy for data handling, scipy for inferential procedures, and scikit-learn and statsmodels for clustering and predictive modeling. Tests were two-sided, uncertainty was expressed as 95% confidence intervals, and results were rounded to three decimals, with p values reported to three decimals and values smaller than 0.001 reported as p < 0.001. Upper- and lower-limb power constructs were defined *a priori* from bench press average power and medicine-ball throw, and from countermovement jump, squat jump, drop jump, and standing long jump, respectively. Indicators were standardized using pre-intervention means and standard deviations, principal component analysis was fitted at baseline separately for each domain with one retained component, and fixed baseline loadings were applied to compute composite scores at pre, mid, and post assessments; responsiveness was quantified as ΔUpper (Post–Pre) and ΔLower (Post–Pre).

Between-group inference for responsiveness used permutation-based procedures to minimize distributional assumptions. For each pairwise contrast in ΔUpper and ΔLower, 100,000 label permutations generated the null distribution of mean differences and yielded two-sided p values, while 20,000 bootstrap resamples produced percentile 95% confidence intervals for mean differences. Effect sizes were expressed as Hedges g with small-sample correction, and Holm adjustment controlled family-wise error across the three pairwise contrasts within each endpoint. Responder signatures were identified by agglomerative hierarchical clustering with Ward’s minimum-variance linkage (Ward, 1963) applied to the standardized two-dimensional change vector (ΔUpper, ΔLower), considering k = 2–4 and selecting the solution primarily by silhouette score while avoiding solutions characterized by very small clusters.

Cluster stability was evaluated with 2,000 bootstrap replicates, refitting the clustering model per replicate, assigning all original participants to bootstrap-derived centroids in standardized change space, aligning bootstrap labels to the original solution by maximum-overlap matching, and quantifying agreement using the adjusted Rand index and cluster-wise Jaccard similarity with 95% bootstrap intervals. Association between training group and responder-signature membership was tested with a permutation chi-square procedure based on 100,000 label permutations. Personalization and moderation were evaluated by ridge regression models for ΔUpper and ΔLower using predictors that included training group indicators, sex, years of training, best 50 m freestyle performance, and baseline composite scores, with continuous predictors standardized and the regularization parameter selected by leave-one-out cross-validation. Cross-validation was used to choose the penalty strength that best generalizes to new swimmers from the same population (reducing overfitting in a small sample) and provide an internal estimate of practical predictive utility. In practical terms, the cross-validated RMSE approximates the typical prediction error (in composite units) for an unseen swimmer, while the cross-validated *R* ^2^ quantifies how much inter-individual variability in responsiveness the model can explain out-of-sample. Model performance was summarized using leave-one-out RMSE and cross-validated *R* ^2^, and coefficient uncertainty was quantified by bootstrap intervals and bootstrap-based two-sided p values. Regularized regression was used because the sample size was small relative to the candidate predictor set and several predictors were expected to be correlated, conditions under which unpenalized models can yield unstable coefficients and inflated apparent effects (Pavlou et al., 2016; Van Calster et al., 2020). L2 shrinkage (ridge) improves numerical stability and reduces overfitting, thereby supporting more reliable out-of-sample prediction under cross-validation (Pavlou et al., 2016; Van Calster et al., 2020). Responder-signature membership was additionally modeled using L2-regularized logistic models with regularization selected by cross-validated log-loss, performance summarized by leave-one-out accuracy and mean log-loss, and uncertainty quantified by bootstrap intervals for log-odds contrasts, with model-based predicted probabilities by training group computed at mean covariate values.

## 3 Results

### 3.1 Construct-level power adaptations across training modalities

Principal component analysis supported a unidimensional upper-limb composite and a unidimensional lower-limb composite. For upper-limb power, the first component explained 0.957 of baseline variance, with equal positive loadings for bench press power and medicine-ball throw (Table 1). The baseline correlation between standardized bench press power and medicine-ball throw was r = 0.913. For lower-limb power, the first component explained 0.949 of baseline variance, with positive loadings across CMJ, SJ, DJ, and SLJ (Table 1).

**TABLE 1:** Principal component loadings for the upper-limb and lower-limb power composites derived from baseline standardized indicators.

Construct	Indicator	Loading (PC1)
Upper-limb power (PC1)	Bench press average power (Pre)	0.707
Upper-limb power (PC1)	Medicine-ball throw (Pre)	0.707
Lower-limb power (PC1)	CMJ (Pre)	0.508
Lower-limb power (PC1)	SJ (Pre)	0.507
Lower-limb power (PC1)	DJ (Pre)	0.509
Lower-limb power (PC1)	SLJ (Pre)	0.475

**FIGURE 1:**
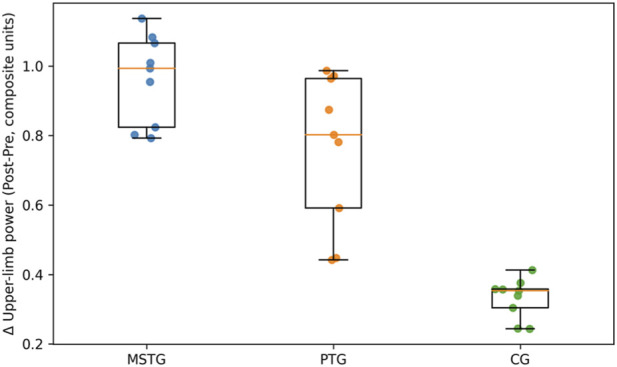
Upper-limb power composite values at Pre, Mid, and Post by training group. Boxplots summarize group distributions at each time point (median and interquartile range), points represent individual swimmers, and lines connect repeated observations within swimmers to visualize individual trajectories across the intervention.

**FIGURE 2:**
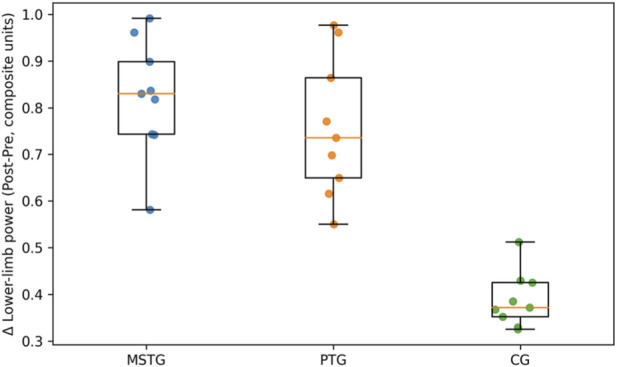
Lower-limb power composite values at Pre, Mid, and Post by training group. Boxplots summarize group distributions at each time point (median and interquartile range), points represent individual swimmers, and lines connect repeated observations within swimmers to visualize individual trajectories across the intervention.

Construct values at pre, mid, and post testing are summarized visually in Figures 1, 2 using side-by-side group-wise boxplots (Pre/Mid/Post) with individual data points (and within-swimmer trajectories), which allows simultaneous appraisal of central tendency, dispersion, and heterogeneity across time, and descriptive statistics for overall construct responsiveness are presented in Table 2.

**TABLE 2:** Descriptive statistics for overall construct responsiveness expressed as delta Post-Pre (composite units).

Endpoint	Group	n	Mean	SD	95% CI
ΔUpper (Post-Pre)	MSTG	9	0.962	0.129	[0.863, 1.061]
ΔUpper (Post-Pre)	PTG	9	0.762	0.218	[0.595, 0.930]
ΔUpper (Post-Pre)	CG	9	0.332	0.058	[0.288, 0.377]
ΔLower (Post-Pre)	MSTG	9	0.822	0.125	[0.726, 0.918]
ΔLower (Post-Pre)	PTG	9	0.758	0.150	[0.643, 0.873]
ΔLower (Post-Pre)	CG	9	0.388	0.059	[0.343, 0.434]

Between-group inference for overall construct responsiveness is summarized in Table 3 and Figures 1, 2 visualize the corresponding Pre–Mid–Post distributions by group, including individual trajectories, providing a graphical complement to Table 3 by showing the time-course of change and the degree of inter-individual variability within each modality. Figures 1, 2 indicates that group separation is already apparent by Mid for both constructs, while the spread of individual trajectories highlights meaningful within-group heterogeneity that is not captured by group means alone.

**TABLE 3:** Pairwise contrasts for overall construct responsiveness using permutation tests, bootstrap 95% confidence intervals for mean differences, and Hedges g effect sizes.

Endpoint	Contrast	Mean difference	95% CI	p (Holm)	Hedges g
ΔUpper (Post-Pre)	MSTG vs. PTG	0.200	[0.047, 0.356]	p = 0.030	1.065
ΔUpper (Post-Pre)	MSTG vs. CG	0.630	[0.543, 0.716]	p < 0.001	6.024
ΔUpper (Post-Pre)	PTG vs. CG	0.430	[0.288, 0.563]	p < 0.001	2.573
ΔLower (Post-Pre)	MSTG vs. PTG	0.065	[-0.056, 0.182]	p = 0.331	0.445
ΔLower (Post-Pre)	MSTG vs. CG	0.434	[0.347, 0.516]	p < 0.001	4.232
ΔLower (Post-Pre)	PTG vs. CG	0.369	[0.274, 0.471]	p < 0.001	3.089

For ΔUpper (Post-Pre), MSTG vs. PTG yielded a mean difference of 0.200 with a 95% confidence interval of [0.047, 0.356], p = 0.030, and Hedges g = 1.065. For ΔUpper (Post-Pre), MSTG vs. CG yielded a mean difference of 0.630 with a 95% confidence interval of [0.543, 0.716], p < 0.001, and Hedges g = 6.024. For ΔUpper (Post-Pre), PTG vs. CG yielded a mean difference of 0.430 with a 95% confidence interval of [0.288, 0.563], p < 0.001, and Hedges g = 2.573.

For ΔLower (Post-Pre), MSTG vs. PTG yielded a mean difference of 0.065 with a 95% confidence interval of [-0.056, 0.182], p = 0.331, and Hedges g = 0.445. For ΔLower (Post-Pre), MSTG vs. CG yielded a mean difference of 0.434 with a 95% confidence interval of [0.347, 0.516], p < 0.001, and Hedges g = 4.232. For ΔLower (Post-Pre), PTG vs. CG yielded a mean difference of 0.369 with a 95% confidence interval of [0.274, 0.471], p < 0.001, and Hedges g = 3.089.

### 3.2 Responder signatures using the two-dimensional change vector defined by ΔUpper (Post-Pre) and ΔLower (Post-Pre)

**FIGURE 3:**
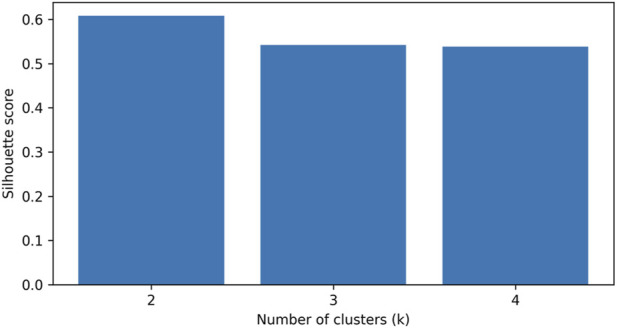
Silhouette scores for candidate solutions (k = 2–4) derived from hierarchical Ward clustering of the standardized two-dimensional change vector (ΔUpper, ΔLower).

Cluster-number selection identified k = 2 as the preferred solution, with silhouette scores and cluster-size diagnostics summarized in Table 4 and shown graphically in Figure 3.

**TABLE 4:** Cluster-number selection metrics for hierarchical Ward clustering applied to the standardized two-dimensional change vector (ΔUpper, ΔLower).

k	Silhouette	Min cluster size	Cluster sizes
2	0.608	12	12; 15
3	0.542	3	15; 9; 3
4	0.539	3	7; 8; 3; 9

**FIGURE 4:**
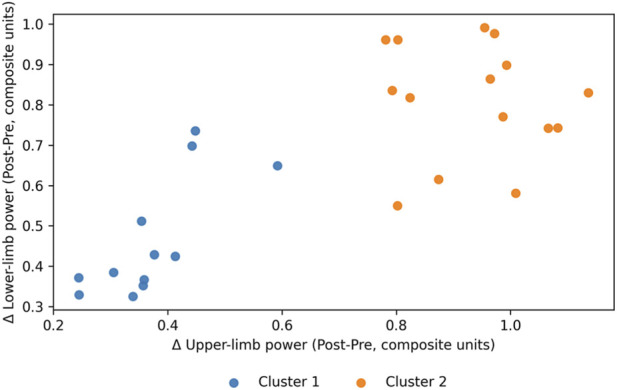
Responder signatures identified by hierarchical Ward clustering (k = 2) in the two-dimensional change space defined by ΔUpper (Post-Pre) and ΔLower (Post-Pre).

The responder signatures for the selected solution (k = 2) are illustrated in the ΔUpper–ΔLower plane in Figure 4, and descriptive cluster profiles are reported in Table 5.

**TABLE 5:** Cluster profiles for responder signatures expressed as ΔUpper (Post-Pre) and ΔLower (Post-Pre) in composite units.

Cluster	Variable	n	Mean	SD	95% CI
1	ΔUpper (Post-Pre)	12	0.373	0.095	[0.312, 0.433]
1	ΔLower (Post-Pre)	12	0.465	0.148	[0.371, 0.559]
2	ΔUpper (Post-Pre)	15	0.936	0.116	[0.872, 1.000]
2	ΔLower (Post-Pre)	15	0.809	0.143	[0.730, 0.889]

**FIGURE 5:**
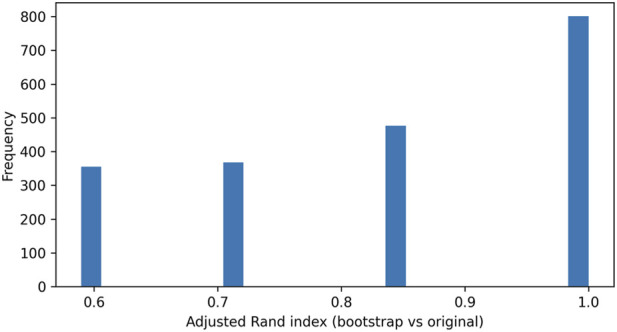
Distribution of adjusted Rand index values obtained from 2,000 bootstrap stability replicates comparing the bootstrap-mapped clustering solution to the original clustering solution. Note: The Rand index quantifies agreement between two partitions based on pairwise co-membership (i.e., whether two swimmers are placed in the same vs. different clusters in each solution), and the ARI is its chance-corrected form; ARI = 1 indicates identical cluster assignments, whereas ARI = 0 indicates agreement no better than expected by chance.

Bootstrap stability analysis indicated overall agreement between the bootstrap-mapped clustering solutions and the original clustering solution, with a mean adjusted Rand index of 0.840 and a 95% bootstrap interval of [0.591, 1.000], with the distribution shown in Figure 5. Because the adjusted Rand index is a chance-corrected measure of agreement between two partitions, a mean ARI of 0.840 indicates high overall reproducibility of the two-cluster solution across bootstrap resamples. The lower bound of the bootstrap interval (0.591) suggests that, even under less favorable resamples, agreement remained moderate-to-high rather than collapsing toward chance. Cluster-wise stability estimates expressed as Jaccard similarity are presented in Table 6. In the present data, mean Jaccard values of 0.907 (Cluster 1) and 0.933 (Cluster 2), with 95% bootstrap intervals of [0.750, 1.000] and [0.833, 1.000], respectively, indicate high cluster-wise stability. Using commonly applied interpretive cut-points (<0.6 unstable, 0.6–0.75 moderate, >0.85 highly stable), both responder signatures can be considered highly stable, with Cluster 2 showing slightly greater reproducibility.

**TABLE 6:** Cluster stability metrics based on 2,000 bootstrap replicates, reported as cluster-wise Jaccard similarity with 95% bootstrap intervals.

Cluster	Cluster size (n)	Mean jaccard	SD jaccard	95% CI
1	12	0.907	0.094	[0.750, 1.000]
2	15	0.933	0.064	[0.833, 1.000]

The Jaccard similarity index quantifies cluster-level reproducibility as the overlap between the members of an original cluster and the most similar cluster recovered in each bootstrap replicate, computed as ∣A∩B∣/∣A∪B∣ , where values closer to 1 indicate that the same swimmers are consistently re-assigned together and values closer to 0 indicate little overlap.

Cluster membership by training group is summarized in Table 7, and the association between group assignment and responder signature was evaluated using a permutation chi-square test with 100,000 label permutations, yielding χ^2^ = 18.900 and p < 0.001, notably, cluster membership closely tracked training allocation (all MSTG swimmers in Cluster 2 and all CG swimmers in Cluster 1), indicating that the two-cluster solution primarily reflects stimulus-dependent response profiles in ΔUpper–ΔLower space rather than training-independent biological responder types.

**TABLE 7:** Distribution of responder signatures (clusters) across training groups.

Cluster	MSTG	PTG	CG
1	0	3	9
2	9	6	0

Cluster membership aligned strongly with training allocation. Thus, clusters should be interpreted as stimulus-dependent response profiles rather than intrinsic responder phenotypes independent of modality.

Personalization and moderation by modeling individual responsiveness and responder-signature membership as functions of baseline characteristics and training modality can be observed in Supplementary Material 1.

## 4 Discussion

The present study examined construct-level power adaptations and stimulus-dependent response profiles (“responder signatures”) following distinct dry-land training approaches in competitive swimmers. The main findings were that maximal strength training produced the largest improvements in the upper-limb power construct and both experimental training approaches exceeded the control condition for upper- and lower-limb responsiveness; and that swimmers segregated into two stable response profiles in the ΔUpper–ΔLower change space with a strong association between training modality and cluster membership, with the observed separation largely reflecting assigned training stimulus (i.e., group allocation accounted for most of the cluster structure) rather than indicating training-independent responder phenotypes.

The first main finding was that upper-limb responsiveness was greater in maximal strength training than plyometric training, and both exceeded the control condition, while lower-limb responsiveness was increased in both training approaches compared with control but did not clearly differ between maximal strength and plyometrics. However, interpretation must be related to the intervention dose and participant level (Tier 2; ∼3.5 h·wk^-1^ swimming). In such samples, large and rapid improvements are plausibly amplified by novelty, task familiarization, and early neural/motor-learning adaptations that predominate in the initial weeks of structured resistance/power training, whereas higher-tier swimmers with extensive, consistent strength-and-conditioning exposure would be expected to show smaller relative changes over the same time window and potentially require longer or higher-dose blocks to shift comparable constructs. Our results are consistent with recent controlled evidence in swimmers indicating that maximal strength training and plyometric training can both enhance sprint-relevant outcomes, with maximal strength training often yielding comparatively larger improvements in strength-dominant or force-dominant outputs (Amara et al., 2021). Broader syntheses in competitive swimming similarly report that combining swim training with structured strength training tends to improve performance more than swim-only approaches, although the magnitude of transfer depends on training content, athlete level, and the specificity of the dry-land stimulus (Fone and van den Tillaar, 2022; Wirth et al., 2022). The comparatively larger upper-limb composite benefits in the maximal strength condition are plausibly explained by neural and contractile adaptations characteristic of heavy strength training, including increases in efferent neural drive, improved voluntary activation, and altered motor unit recruitment and rate coding, which manifest early and are particularly relevant for high-force actions that underpin propulsive phases (Sale, 1988; Del Vecchio et al., 2019; Tøien et al., 2021). The robust improvements in the lower-limb construct across both training interventions align with the shared capacity of maximal strength and plyometrics to enhance force–velocity characteristics and stretch–shortening cycle performance, which are central to jump-derived outcomes and are strongly implicated in swimming start and turn performance (Potdevin et al., 2011; West et al., 2011; Keiner et al., 2021).

The second main finding was the emergence of two stimulus-dependent response profiles in ΔUpper–ΔLower space, characterized by a lower-change cluster and a higher-change cluster, with good bootstrap stability and a pronounced association between training group and responder category; indeed, cluster membership closely mirrored training allocation (MSTG concentrated in the higher-change cluster and CG concentrated in the lower-change cluster), implying that the “responder signatures” primarily summarize between-group stimulus effects rather than intrinsic, modality-independent responder biology. These signatures are defined specifically in the ΔUpper–ΔLower change space (i.e., the constructs as operationalized in this study) rather than implying global responder “types.” This aligns with a growing body of work showing substantial inter-individual response variability to standardized training and that “responder” classification is sensitive to the outcomes selected, measurement error, and the analytic method used to form subgroups (including clustering) (Potdevin et al., 2011; Basri and Turki, 2025). Within swimming-specific contexts, controlled trials and syntheses of dry-land modalities indicate that the imposed training stimulus can materially influence both the magnitude and heterogeneity of performance-related changes, which is consistent with (though not definitive proof of) stimulus-dependent responder structure (Lopes et al., 2021). Two-cluster structure in ΔUpper–ΔLower space is consistent with the idea that adaptation is constrained by the magnitude of the imposed neuromuscular stimulus and (within a given stimulus) inter-individual differences in training dose uptake, fatigue–recovery balance, and motor learning (Mann et al., 2014). However, in the present dataset the dominant determinant of cluster membership was the assigned training stimulus, and disentangling intrinsic individual factors from modality effects will require designs with larger samples, broader outcomes (including in-water transfer), and/or clustering within-modality strata. In practical terms, the high stability estimates observed for both clusters suggest that the responder signatures were not merely stochastic partitions of noise but reflected reproducible patterns in the multivariate change signal.

In practical terms, the high stability estimates observed for both clusters suggest that the responder signatures were not merely stochastic partitions of noise but reflected reproducible patterns in the multivariate change signal. The dominance of the training stimulus accords with the evidence base in swimming indicating that targeted dry-land programs, particularly those emphasizing heavy strength or well-structured power training, are meaningful determinants of start, turn, and sprint-related outcomes, which are strongly influenced by maximal and explosive strength capacities (West et al., 2011; Keiner et al., 2021). Importantly, in a brief, low-dose block delivered to trained/developmental training group dominated the ridge/logistic models indicates that the ‘predictive’ structure is driven primarily by between-group stimulus allocation, with limited incremental personalization signal from baseline covariates in this context. The inverse association between baseline sprint performance and high-responder probability is consistent with ceiling effects and diminishing returns that are especially plausible in lower-trained or highly trainable samples over short interventions, where swimmers with higher initial status may show smaller observable changes because they operate closer to performance limits and require larger or more prolonged stimuli to shift outcomes (Mann et al., 2014; Bonafiglia et al., 2016). Accordingly, these predictive findings should be interpreted as conditional on participant level and intervention duration, and should not be generalized to athlete monitoring, talent development, or higher-tier swimmers without external validation in independent cohorts and longer training periods. A complementary explanation is that better baseline sprint performance may reflect greater technical proficiency and efficiency, reducing the relative contribution of dry-land power gains to the measured composite changes and thereby attenuating the apparent responsiveness signal, a phenomenon previously discussed in the swimming literature as limited transfer when dry-land gains do not align with in-water coordination and propulsive specificity (Wirth et al., 2022).

Some limitations should be considered. First, although eligibility required at least 2 years of resistance-training experience, we did not quantify participants’ prior dry-land strength and conditioning exposure or verify consistency with training logs. Baseline training status and familiarity with barbell versus jump/medicine-ball tasks may therefore have differed, making it difficult to disentangle modality-specific adaptations from generalized responses to the introduction of a supervised, progressive program. Second, while the use of composite constructs strengthens reliability and reduces multiplicity, it necessarily compresses information and may mask indicator-specific adaptation profiles, particularly if different neuromuscular qualities contribute heterogeneously across tests. This issue is compounded by partial overlap between training content and outcome measures, which can introduce specificity/learning bias. Specifically, bench press average power contributed to ΔUpper and was directly trained only in MSTG, whereas ΔLower comprised jump-based tests (CMJ, SJ, DJ, SLJ) that closely resemble exercises emphasized in PTG. Therefore, between-group differences, especially the MSTG advantage for ΔUpper, should be interpreted primarily as superiority in trained-task responsiveness rather than unequivocal evidence of greater generalized power transfer. Future trials should minimize exercise–outcome overlap (or pre-specify both trained-task and transfer endpoints) and prioritize outcomes with clearer in-water relevance (e.g., start/turn measures, in-water kinetics, and race performance). Finally, although clustering stability was formally evaluated, responder classification remains sensitive to feature choice and scaling and should be examined in larger multi-center samples. Importantly, the relatively small sample size limits generalizability and constrained longitudinal modeling, whereas larger samples would enable linear mixed-effects models for repeated measures, which are generally preferred for longitudinal data and can better accommodate individual trajectories and missing observations. While plyometric sessions were closely supervised and standardized with SSC-focused coaching cues, we did not collect objective kinematic or ground contact-time metrics during training; thus, we cannot confirm the extent to which all participants consistently executed a fast SSC across sessions, which may have introduced unquantified variability in the realized PTG stimulus. Additionally, because cluster membership aligned strongly with training allocation in this sample, the identified “responder signatures” should be interpreted primarily as stimulus-dependent response profiles (between-group structure) rather than training-independent individual phenotypes; future work should test whether similar profiles emerge within-modality, across longer periods, and using outcomes with clearer in-water transfer. In addition, the intervention was brief and low-frequency (12 sessions across 6 weeks) and the sample was trained/developmental with modest in-water volume (∼3.5 h·wk^-1^), which likely increased short-term trainability and novelty effects; therefore, the magnitude and speed of the observed adaptations should not be assumed to generalize to higher-tier (Tier 3–5) or elite swimmers with higher training loads and longer strength-training histories.

From an applied perspective, in Tier 2 (trained/developmental) university swimmers completing a short, low-dose dry-land block (12 sessions/6 weeks), the present findings suggest that introducing supervised, progressive dry-land training, whether maximal strength–oriented or plyometric, can yield meaningful short-term improvements in the measured power constructs, with maximal strength training producing a larger increase in the upper-limb construct as operationalized here. Because responsiveness in this setting is likely amplified by high trainability and novelty/familiarization effects, these data should not be used to make strong preferential prescriptions for higher-tier or elite swimmers, for whom marginal gains over short blocks are typically smaller and more dose-dependent. Practitioners may therefore view MSTG and PTG as context-dependent options within periodized plans, and prioritize monitoring of individual change (ΔUpper/ΔLower) while integrating outcomes with in-water performance transfer (starts/turns and race metrics) when making programming decisions. However, any model-based ‘prediction’ shown here should be treated as exploratory.

## 5 Conclusion

Maximal strength training elicited the greatest improvement in the upper-limb composite construct (ΔUpper; bench press average power and medicine-ball throw), while both maximal strength and plyometric training improved upper- and lower-limb composite constructs relative to the control condition. Because the outcome constructs partially overlapped with the training content (bench press power in MSTG; jump-based tests in PTG), between-modality differences, particularly for ΔUpper, should be interpreted as construct-/trained-task responsiveness that may be influenced by specificity/learning effects, rather than as definitive evidence of generalized neuromuscular superiority or a basis for strong preferential recommendations. Swimmers also displayed two stable responder signatures in ΔUpper–ΔLower change space that were strongly associated with training modality. However, these responder signatures should be interpreted as modality-aligned patterns within this short intervention and sample, rather than as definitive evidence of baseline-determined “responder” profiles. Therefore these predictive findings should be viewed as context-conditional and not generalized without external validation. Larger and longer-duration trials that better quantify/equate training dose across modalities and incorporate in-water transfer outcomes are needed to confirm modality-specific transfer to swimming performance.

## Data Availability

The raw data supporting the conclusions of this article will be made available by the authors, without undue reservation.
