# Supplementary material for: Responder signatures and predictors of upper- and lower-limb power responsiveness to maximal strength versus plyometric dry-land training in swimmers
Source: Front Physiol. 2026 Mar 4;17:1799253. doi: 10.3389/fphys.2026.1799253 (PMC12995655; doi:10.3389/fphys.2026.1799253)
Supplement: Supplementary file 1 [file DataSheet1.pdf]

Supplementary material 1. Personalization and moderation by modeling individual responsiveness and responder-signature membership as functions of baseline characteristics and training modality

Leave-one-out cross-validation selected the ridge L2 penalty parameter $\lambda = 0.001$ for both ΔUpper and ΔLower. Because our models use Post–Pre change scores (ΔUpper, ΔLower), each swimmer contributes a single observation. Therefore, within-subject repeated-measures clustering is not present in this specific analysis. Coefficient estimates with bootstrap intervals are provided in Tables S1 and S2, where the MSTG and PTG coefficients represent covariate-adjusted mean differences versus the control group (CG). They are expected to be close to, but not identical with, the unadjusted permutation-based mean differences reported in Table 3 because ridge models adjust for baseline covariates and apply coefficient shrinkage to reduce overfitting in small samples. Coefficient estimates with bootstrap intervals are provided in Tables S1 and S2. For unadjusted group contrasts based purely on group means, see Table 3.

S1. Ridge regression coefficients for ΔUpper (Post-Pre) with percentile 95% bootstrap intervals and bootstrap-based two-sided p values. Continuous predictors were standardized. MSTG and PTG coefficients are covariate-adjusted mean differences versus CG (in composite units).

Predictor	Estimate	95% CI	p
Intercept	0.382	[0.091, 0.572]	$p = 0.016$
MSTG	0.628	[0.438, 0.790]	$p < 0.001$
PTG	0.439	[0.180, 0.637]	$p = 0.006$
Sex01	-0.141	[-0.775, 0.813]	$p = 0.702$
YearsTrain_z	0.034	[-0.089, 0.123]	$p = 0.628$
Best50_z	-0.013	[-0.208, 0.199]	$p = 0.750$
Upper_Pre_z	-0.062	[-0.252, 0.211]	$p = 0.512$
Lower_Pre_z	-0.044	[-0.217, 0.154]	$p = 0.610$

S2. Ridge regression coefficients for ΔLower (Post-Pre) with percentile 95% bootstrap intervals and bootstrap-based two-sided p values. Continuous predictors were standardized. MSTG and PTG coefficients are covariate-adjusted mean differences versus CG (in composite units).

Predictor	Estimate	95% CI	p
Intercept	0.412	[0.194, 0.580]	p = 0.002
MSTG	0.408	[0.235, 0.600]	p < 0.001
PTG	0.363	[0.188, 0.511]	p = 0.004
Sex01	-0.035	[-0.614, 0.659]	p = 0.832
YearsTrain_z	-0.007	[-0.077, 0.068]	p = 0.826
Best50_z	-0.038	[-0.160, 0.165]	p = 0.594
Upper_Pre_z	-0.018	[-0.169, 0.188]	p = 0.788
Lower_Pre_z	-0.042	[-0.201, 0.095]	p = 0.546

Table S3. Leave-one-out cross-validated predictive performance for ridge models of ΔUpper and ΔLower.

Outcome	RMSE	R^2	λ
ΔUpper (Post-Pre)	0.194	0.579	0.001
ΔLower (Post-Pre)	0.153	0.523	0.001

Note: Leave-one-out cross-validation refits the model while holding out one swimmer at a time. The resulting RMSE and R^2 provide an internal estimate of expected prediction error and explained variance for new swimmers from the same population, and λ is the penalty strength chosen to maximize out-of-sample performance. λ controls L2 shrinkage strength.

Cross-validated predictive performance is summarized in Table S3 and illustrated by observed versus predicted plots in Figures X1 and X2. The leave-one-out estimates indicate the expected out-of-sample accuracy of the personalization models. R^2 values around 0.52–0.58 suggest moderate explainability of responsiveness, while the RMSE values (0.15–0.19 composite units) reflect non-trivial residual variability, implying that individual-level predictions should be interpreted with caution even when group effects are present.

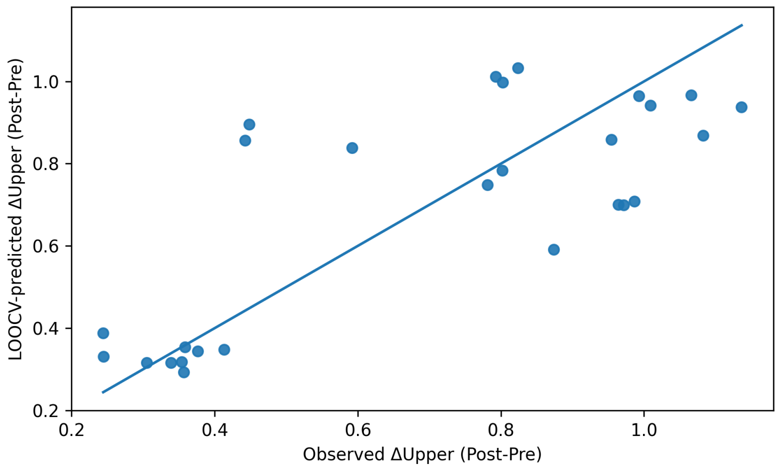

Figure X1. Observed versus leave-one-out cross-validated predicted values for ΔUpper (Post-Pre) from ridge regression. The diagonal line indicates identity.

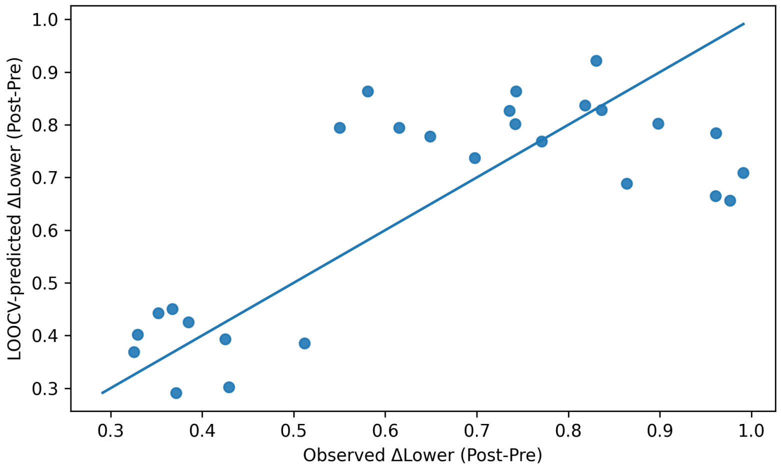

Figure X2. Observed versus leave-one-out cross-validated predicted values for ΔLower (Post-Pre) from ridge regression. The diagonal line indicates identity.

Model-based marginal predicted responsiveness by training group at mean covariate values is presented in Table S4, providing an adjusted interpretation of group effects within the personalization models.

Table S4. Model-based marginal predicted mean responsiveness by training group at mean covariate values, with percentile 95% bootstrap intervals.

Outcome	Group	Predicted mean	95% CI
ΔUpper	CG	0.330	[0.224, 0.457]
ΔUpper	MSTG	0.958	[0.829, 1.079]
ΔUpper	PTG	0.769	[0.581, 0.921]
ΔLower	CG	0.399	[0.280, 0.499]
ΔLower	MSTG	0.808	[0.696, 0.915]
ΔLower	PTG	0.762	[0.639, 0.854]

Responder-signature membership for the selected clustering solution (k = 2) was modeled using logistic regression with L2 regularization, selecting C = 3.511 by cross-validated log-loss. Leave-one-out predictive performance yielded an accuracy of 0.741 and a mean log-loss of 0.411. Coefficient estimates with bootstrap intervals are summarized in Table S5, and model-based predicted cluster probabilities by group at mean covariate values are displayed in Figure X3.

Table S5. Logistic regression coefficients for responder-signature membership reported as log-odds differences with percentile 95% bootstrap intervals and bootstrap-based two-sided p values.

Comparison	Predictor	Estimate	95% CI	p
Cluster 2 vs Cluster 1	Intercept	-1.043	[-1.463, -0.323]	p = 0.025
Cluster 2 vs Cluster 1	MSTG	2.133	[1.354, 2.465]	p < 0.001
Cluster 2 vs Cluster 1	PTG	1.193	[0.261, 1.919]	p = 0.010
Cluster 2 vs Cluster 1	Sex01	0.428	[-0.206, 1.074]	p = 0.205
Cluster 2 vs Cluster 1	YearsTrain_z	0.227	[-0.292, 0.744]	p = 0.345
Cluster 2 vs Cluster 1	Best50_z	-0.550	[-1.382, -0.052]	p = 0.030
Cluster 2 vs Cluster 1	Upper_Pre_z	0.381	[-0.429, 1.056]	p = 0.345
Cluster 2 vs Cluster 1	Lower_Pre_z	-0.459	[-1.176, 0.205]	p = 0.190

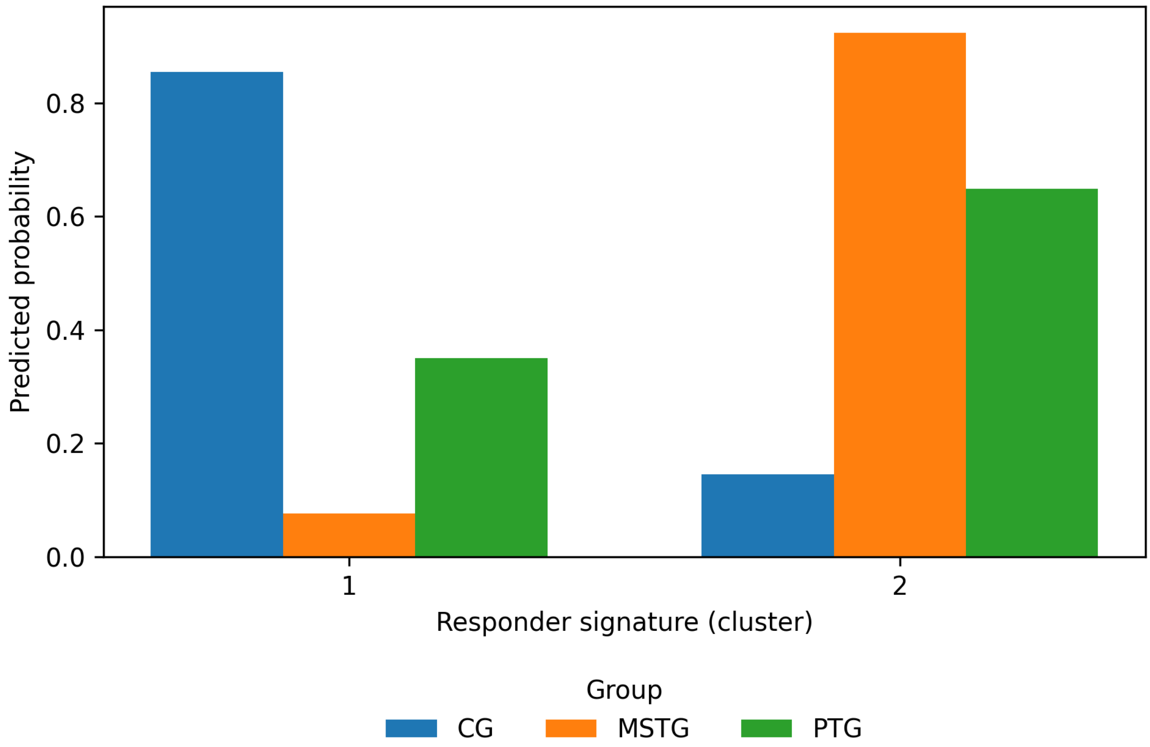

Figure X3. Model-based predicted probabilities of responder-signature membership by training group at mean covariate values from logistic regression.
